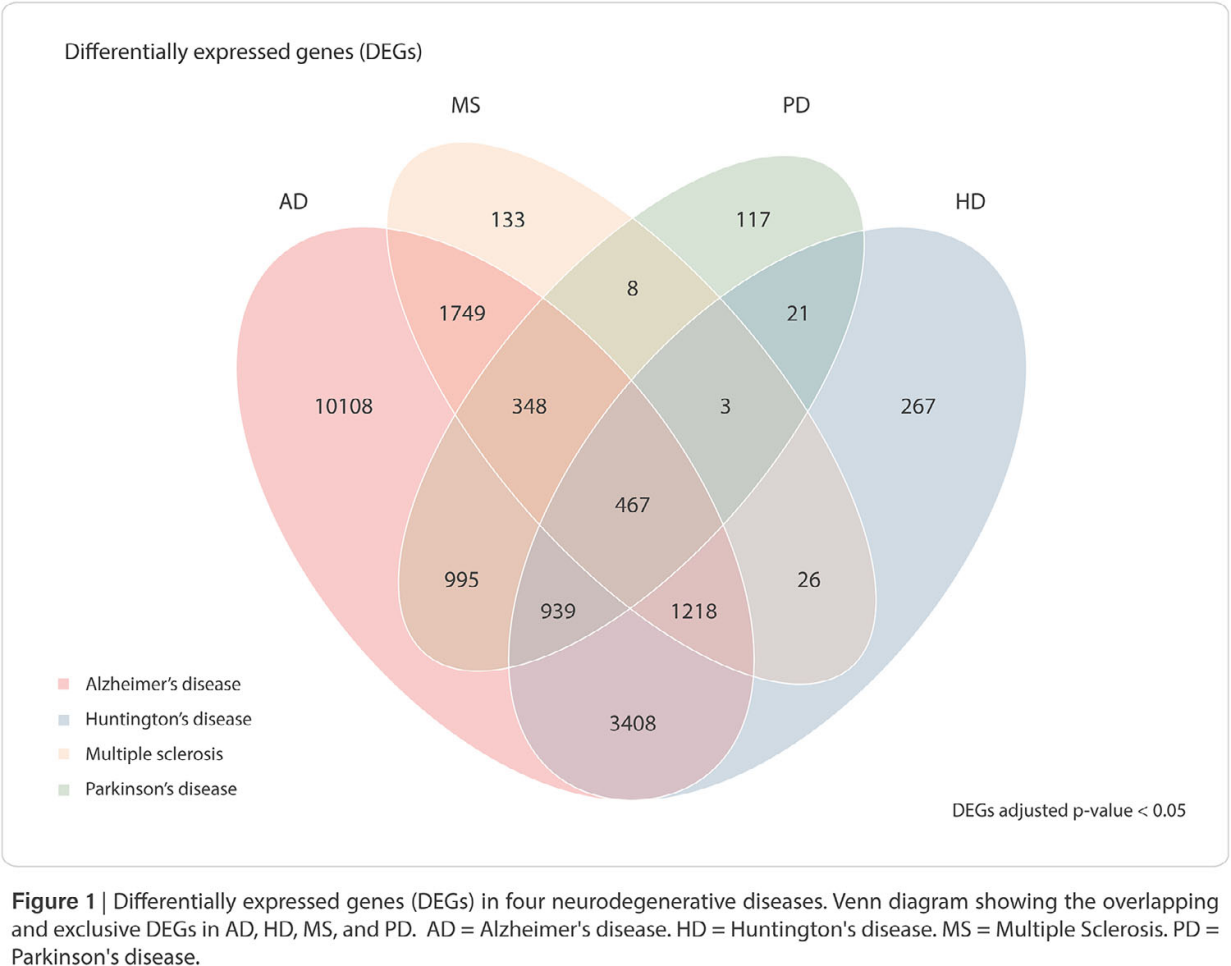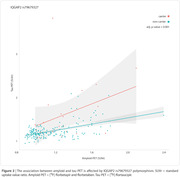# A single nucleotide polymorphism from a differentially‐expressed gene shared across four neurodegenerative diseases increases tau accumulation independently of amyloid

**DOI:** 10.1002/alz.091792

**Published:** 2025-01-09

**Authors:** Vanessa G. Ramos, Giovanna Carello‐Collar, Thomas Hugentobler Schlickmann, Marina Siebert, Alexandre Santos Cristino, Marco De Bastiani, Eduardo R. Zimmer

**Affiliations:** ^1^ Universidade Federal do Rio Grande do Sul, Porto Alegre, Rio Grande do Sul Brazil; ^2^ Universidade Federal do Rio Grande do Sul, Porto Alegre, RS Brazil; ^3^ Clinical Hospital of Porto Alegre, Porto Alegre, Rio Grande do Sul Brazil; ^4^ Griffith Institute for Drug Discovery, Griffith University, Brisbane Australia

## Abstract

**Background:**

Neurodegenerative diseases (NDs), including Alzheimer's disease (AD), Huntington's disease (HD), Multiple Sclerosis (MS), and Parkinson's disease (PD) are characterized by the accumulation of misfolded proteins and progressive loss of neurons. However, whether a neurodegeneration transcriptomic signature exists remains uncertain. Thus, we aimed to explore differentially expressed genes (DEGs) in common to AD, HD, MS, and PD. We also seek to evaluate the effect of single nucleotide polymorphisms (SNPs) derived from the NDs‐shared DEGs on AD pathophysiology.

**Methods:**

We obtained RNA‐sequencing and microarray datasets of vulnerable brain regions of AD, HD, MS, and PD individuals from the Gene Expression Omnibus. Differential gene expression analysis (adjusted p‐value < 0.05) was conducted to identify DEGs in common among these four NDs. Then, we examined the impact of shared DEG‐related SNPs on amyloid and tau burden in AD through a linear regression model adjusted for the covariates of age, sex, and *ApoE*ε4 status. We obtained DEG‐related SNPs and amyloid‐ and tau‐PET data from the Alzheimer's Disease Neuroimaging Initiative (ADNI).

**Results:**

We identified 467 DEGs in common among AD, HD, MS, and PD vulnerable brain regions (Figure 1, p‐value < 0.05). From these DEGs, 15,574 SNPs were available in ADNI. Linear regression analysis revealed that IQGAP2 rs79679327 carriers had higher tau accumulation independently of amyloid burden (Figure 2, p‐value = 0.001; carriers: n = 21, mean age ± standard deviation (SD) = 72.76 ± 4.54; non‐carriers: n = 176, mean age ± SD = 71.64 ± 6.85).

**Conclusion:**

Here, we show transcriptional similarities across four NDs. In AD, individuals carrying the IQGAP2 rs79679327 variant exhibited greater tau accumulation, regardless of amyloid. Our findings may provide insights into neurodegenerative traits, through a common signature of neurodegeneration.